# Supplementary material for: BIN overlap confirms transcontinental distribution of pest aphids (Hemiptera: Aphididae)
Source: PLoS One. 2019 Dec 10;14(12):e0220426. doi: 10.1371/journal.pone.0220426 (PMC6903727; doi:10.1371/journal.pone.0220426)
Supplement: Attachment — Submitted filename: Response-to-Reviewers_PONE-D-19-19598R1.docx [file pone.0220426.s006.docx]

November 19, 2019

PONE-D-19-19598: BIN overlap confirms transcontinental distribution of pest aphids (Hemiptera: Aphididae).

**Response to Reviewers**

The comments from the Reviewers are included in italics followed by our response.

**Reviewer Comments to Author:**

**Reviewer #2:**

*COMMENT: It is nice to see that the authors accepted most comments and/or remarks. Some minor concerns still remain (see sticky notes). After correction, the manuscript should be suitable for publication in PLoS ONE.*

Response: All the comments, pointed out through sticky notes on the pdf, have been addressed in the revised version.

*COMMENT: L137: Which program was used? MUSCLE? You should mention it (inc. giving the reference).*

Response: Nucleotide sequence alignment was performed using ClustalW. This has been mentioned, with relevant reference, in the revised version.

*COMMENT: L299: Most DOIs and PMIDs are still missing*

Response: We have followed the reference style for PLOS ONE. This style does not ask for DOIs and PMIDs when the volume and page numbers are available.

**Reviewer #3:**

*COMMENT: I like that the authors have carefully addressed both the issues that I myself spotted, but also the issues spotted by the other reviewers. I noticed that one of my own comments was with a mistake. I definitely did not mean to change the word "exploitation" to "expointation" in the reference list, but to remove the double capital in the previous word (EXploitation > Exploitation).*

Response: The comment has been addressed.
